# Supplementary material for: Altered microRNA Transcriptome in Cultured Human Liver Cells upon Infection with Ebola Virus
Source: Int J Mol Sci. 2021 Apr 6;22(7):3792. doi: 10.3390/ijms22073792 (PMC8038836; doi:10.3390/ijms22073792)
Supplement: Supplementary file 1 [file ijms-22-03792-s001.zip › Supplementary_File/D_ KEGG_Analysis_Results/16-30nt_pathway_Makona-24h-Huh7_vs_Control-24h-Huh7_down.mature_mirna_targets/hsa_EnrichmentScoreDotPlot.pdf]

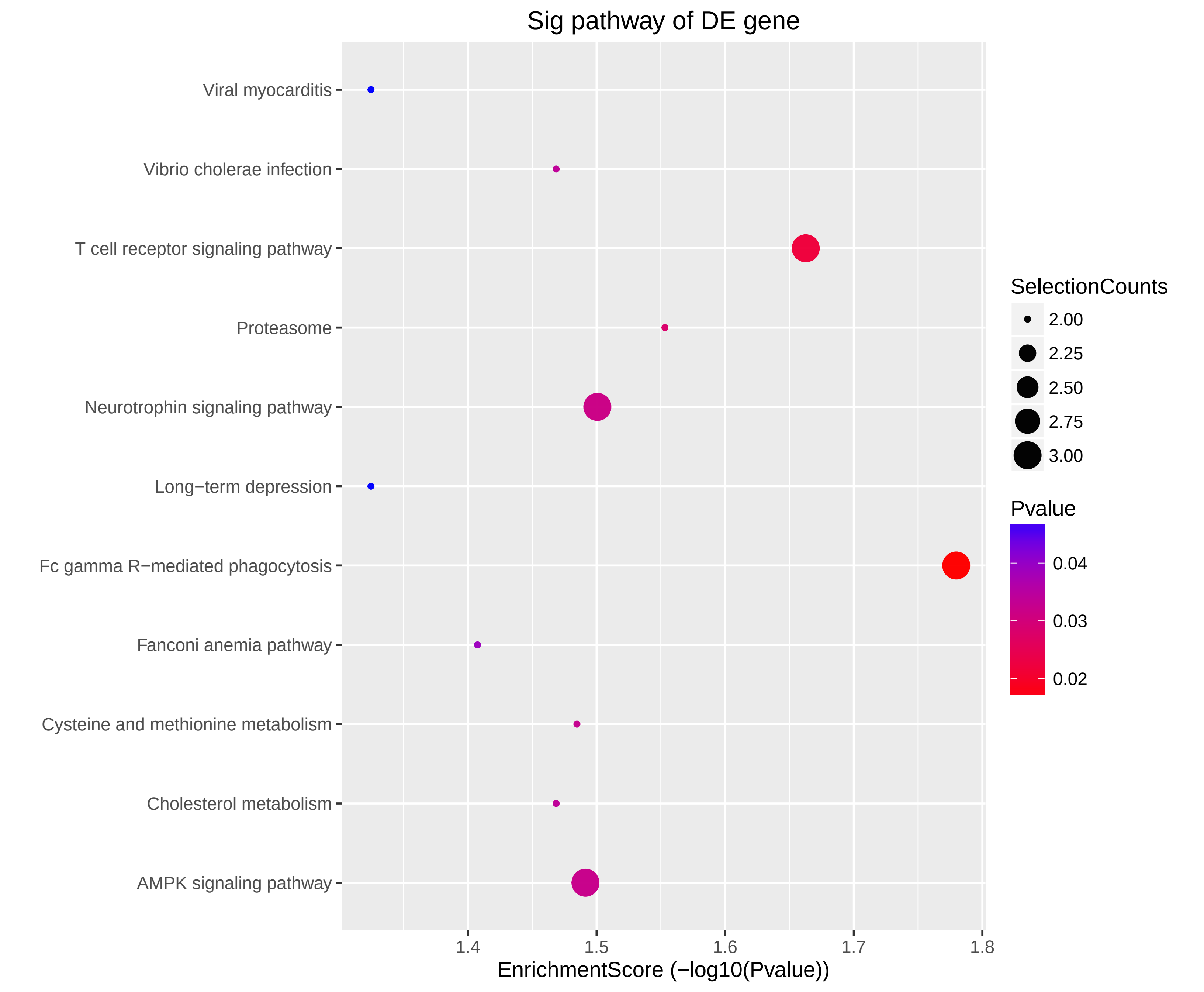
Sig pathway of DE gene
Viral myocarditis
Vibrio cholerae infection
T cell receptor signaling pathway
Proteasome
Neurotrophin signaling pathway
Long−term depression
Fc gamma R−mediated phagocytosis
Fanconi anemia pathway
Cysteine and methionine metabolism
Cholesterol metabolism
AMPK signaling pathway
1.4
1.5
1.6
1.7
1.8
EnrichmentScore (−log10(Pvalue))
SelectionCounts
2.00
2.25
2.50
2.75
3.00
Pvalue
0.04
0.03
0.02
